# Formose Reaction Controlled by a Copolymer of *N*,*N*-Dimethylacrylamide and 4-Vinylphenylboronic Acid

**DOI:** 10.3390/polym9110549

**Published:** 2017-10-25

**Authors:** Tomohiro Michitaka, Toru Imai, Akihito Hashidzume

**Affiliations:** Department of Macromolecular Science, Graduate School of Science, Osaka University, Osaka 560-0043, Japan; michitakat12@chem.sci.osaka-u.ac.jp (T.M.); imi.t0.ru@gmail.com (T.I.)

**Keywords:** formose reaction, boronic acid compounds, *N*,*N*-dimethylacrylamide/4-vinylphenylboronic acid copolymer, phenylboronic acid, monosaccharides, sugar alcohols

## Abstract

The formose reaction is an oligomerization of formaldehyde under basic conditions, which produces a complicated mixture of monosaccharides and sugar alcohols. Selective formation of useful monosaccharides by the formose reaction has been an important challenge. In this study, we have investigated the formose reaction controlled by *N*,*N*-dimethylacrylamide/4-vinylphenylboronic acid copolymer (pDMA/VBA) and phenylboronic acid (PBA) because boronic acid compounds form esters with polyols, e.g., monosaccharides and sugar alcohols. We obtained time–conversion data in the presence of these boronic acid compounds, and characterized the products by liquid chromatography-mass spectroscopy and NMR measurements. pDMA/VBA and PBA decelerated the formose reaction because of the formation of boronic acid esters with products. It is noteworthy that the formose reaction in the presence of pDMA/VBA and PBA formed favorably six- and seven-carbon branched monosaccharides and sugar alcohols.

## 1. Introduction

Carbohydrates are ubiquitous in our life and very important biological materials [1,2]. Carbohydrates can be divided into three categories based on their molecular weight; (1) low molecular weight saccharides, e.g., monosaccharides and disaccharides, (2) oligosaccharides, and (3) polysaccharides. Low molecular weight saccharides are utilized as energy source for living organisms, and oligosaccharides exhibit a number of physiological activities. Two representative polysaccharides are cellulose and starch; the former is utilized as a structural material in plants whereas the latter is employed as energy storage. Carbohydrates used in biological systems are synthesized by enzymatic reactions [1,2]. Synthesis of carbohydrates in a non-enzymatic way has been an important subject of investigation [3,4,5,6]. Especially, synthesis of monosaccharides from starting materials containing one or two carbon atoms is still remaining as a challenging subject because monosaccharides possess a number of chiral centers to be controlled [7,8].

Since monosaccharides, e.g., glyceraldehyde, erythrose, ribose, and glucose have the general formula (CH_2_O)*_n_*, monosaccharides can be formed by oligomerization of formaldehyde. In 1861, Butlerow [9] reported that a sugar-like product called ‘formose’ is obtained from formaldehyde by heating in water under basic conditions. This reaction is called the formose reaction. Detailed studies have revealed that the formose reaction proceeds through three major stages [10,11,12,13]. In the first stage, two formaldehyde molecules couple to form glycolaldehyde. Since this reaction is the rate-determining step, the formose reaction indicates an induction period. As glycolaldehyde acts as a co-catalyst, a variety of monosaccharides are formed dominantly through aldol reaction and aldose–ketose isomerization in the second stage. This stage is called the sugar-forming period. After completion of conversion of formaldehyde, the monosaccharides formed are decomposed mainly through retro-aldol and Cannizzaro reactions in the third stage. This stage is called the sugar-decomposing period. Thus, the formose reaction yields a complicated mixture of monosaccharides and sugar alcohols through these three stages. The formose reaction is considered as the most promising pathway to form monosaccharides under prebiotic conditions [14,15,16,17,18,19].

If the formose reaction proceeds in a controlled manner, it will provide an important pathway for non-enzymatic synthesis of monosaccharides. Some pioneering works on a selective formose reaction were conducted from the 1970s to the 1980s. Shigemasa et al. [20,21,22,23] devoted their remarkable efforts to optimize the conditions. Consequently, they first isolated a branched seven-carbon sugar alcohol from the reaction mixture of the formose reaction catalyzed by barium hydroxide in methanol [24,25]. Matsumoto et al. [26,27] successfully synthesized 1,3-dihydroxyacetone, a three-carbon monosaccharide, using a thiazolium catalyst in ethanol in the presence of triethylamine. After these pioneering works, however, there have been only a few studies on a selective formose reaction [28,29] and, thus, the selective formation of useful monosaccharides by the reaction remains under-examined.

We have been working on the formose reaction controlled by molecular recognition of polymers and molecular assemblies [30,31,32]. We have reported that the reaction is accelerated in reverse micelles of aerosol-OT, an anionic surfactant, and yields ethylene glycol as a major product [31].

**Scheme 1:**
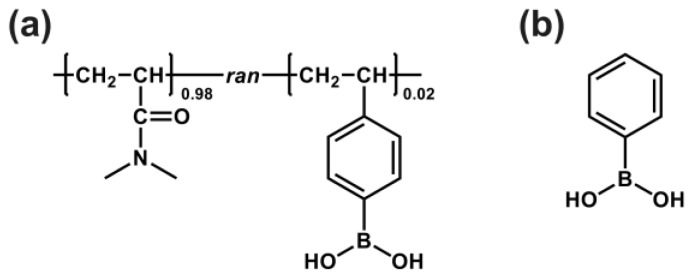
Chemical structures of pDMA/VBA (**a**) and PBA (**b**).

Recently, we have also utilized boronic acid compounds to control the formose reaction because boronic acid compounds form esters with polyols, e.g., monosaccharides or sugar alcohols. This phenomenon has been utilized as artificial molecular sensors for saccharides [33,34,35,36] and as polymeric materials responsive to saccharides [37]. When the formose reaction is carried out in the presence of boronic acid compounds, boronic acid esters may be formed to stabilize the products, leading to an enhanced selectivity. We have, thus, studied the formose reaction in the presence of sodium phenylboronate (NaPB) and an anionic copolymer (pNaSS/NaVB) of sodium 4-styrenesulfonate (NaSS) and sodium 4-vinylphenylboronate (NaVB) [32]. We observed that NaPB and pNaSS/NaVB decelerated the reaction. More importantly, we exhibited that the formose reaction produced three- to five-carbon monosaccharides preferably in the presence of NaPB and four- to eight-carbon sugar alcohols favorably in the presence of pNaSS/NaVB. However, we also observed that NaSS polymer decelerated the formose reaction. We have, thus, been motivated to use other boronic acid polymers. In this article, we describe the formose reaction carried out in the presence of a copolymer of non-ionic *N*,*N*-dimethylacrylamide and 4-vinylphenylboronic acid (pDMA/VBA, Scheme 1).

## 2. Materials and Methods

### 2.1. Materials

Methanol-*d*_4_ (>99.8%, Merck, Darmstadt, Germany), deuterium oxide (D_2_O, 99.9atom%D, Sigma Aldrich, St. Louis, MO, USA), dimethyl sulfoxide-*d*_6_ (DMSO-*d*_6_, 99.5 atm%, Sigma-Aldrich), acetonitrile (>99.8%, HPLC grade, Kishida Chemical, Osaka, Japan), and dimethyl solfoxide (DMSO, >98.0%, Nacalai Tesque, Kyoto, Japan) were purchased and used as received. 4-Vinylbenzyltrimethylammonium chloride (VTAC, 99%, Sigma Aldrich), 4,4′-azobis(4-cyanovaleric acid) (ACVA, 98.0+%, Wako, Osaka, Japan), 4-vinylphenylboronic acid (VBA, >97%, TCI, Tokyo, Japan), calcium hydroxide (Ca(OH)_2_, >95.0%, Sigma Aldrich), phenylboronic acid (PBA, >97.0%, TCI), lithium bromide (>98.0%, Nacalai Tesque), poly(sodium 4-styrenesulfonate) (pNaSS, average *M*_w_~1,000,000, Sigma Aldrich), and Amberlite^®^ IR120 (hydrogen form, Sigma Aldrich) were purchased and used as received. Acetylacetone (99.0+%, Wako), acetic acid (>99.0%, Nacalai Tesque), ammonium acetate (>97.0%, Nacalai Tesque), and ethanol (>99.5%, Shinwa Alcohol) were used for the acetylacetone method as received. *N*,*N*-Dimethylformamide (DMF, >99.5%, Kanto Chemical) was purified using a Glass Contour solvent dispensing system. *N*,*N*-Dimethylacrylamide (DMA) (>99.0%, KJ Chemicals, Tokyo, Japan) was used after treatment with a short alumina column. Water was purified with a Millipore Elix 5 system. Formaldehyde solution (36.0–38.0%, Wako) was diluted with water to adjust 1/12 vol% and used for the formose reaction. Amberlite^®^ IRA410 (chloride form, Sigma Aldrich) was used after treatment with 1 M NaOH, followed by washing with water. Spectra/Por 5 dialysis tubing, 12–14 kD MWCO (Spectrum Laboratories) was used for dialysis. Other reagents were used as received.

### 2.2. Measurements

Size exclusion chromatography (SEC) measurements were performed on a TOSOH HLC-8320GPC EcoSEC equipped with a TOSOH TSK gel α-M column using 1.05 g·L^−1^ lithium bromide in DMSO as the eluent at 40 °C, with a flow rate of 0.4 mL min^−1^. The number of average molecular weights (*M*_n_) and the ratios of the weigtht to the number average molecular weights (*M*_w_/*M*_n_) were calibrated with polyacrylamide standards (American Polymer Standards).

UV-VIS spectra were recorded on a HITACHI U-4100 spectrophotometer equipped with a cell holder thermostated at 25 °C using a Yamato CF300 circulator for the acetylacetone method.

IR spectra were recorded on a JASCO FT/IR-6100 spectrometer equipped with an ATR PRO410-S carrying a diamond prism.

Liquid chromatography-mass spectroscopy (LC-MS) measurements were performed on a Bruker Daltonics micrOTOF-QIII compact connected with a Shimadzu Prominence UFLC system. In the LC system, Shimadzu LC-20AD pumps and a Shodex 5NH2P-50 4E column were equipped, and a mixed solvent of water and acetonitrile (25/75, *v*/*v*) containing 0.1 vol% formic acid was used as an eluent at a flow rate of 0.6 mL min^−1^.

^13^C NMR and distortionless enhancement of polarization transfer with a pulse delay adjusted for 3π/4 (DEPT-135) measurements were performed on a JEOL JNM ECA500 spectrometer using D_2_O as a solvent at 25 °C. Two-dimensional heteronuclear single-quantum correlation (HSQC) and heteronuclear multiple bond correlation (HMBC) spectra were recorded on a Bruker AVANCE700 spectrometer using D_2_O as a solvent at 25 °C. Acetonitrile was used as an internal standard, and chemical shifts were referenced to the signal due to the methyl carbon of acetonitrile (*δ* = 1.47 ppm).

### 2.3. Preparation of Water Soluble Polymers

VTAC (4.23 g, 20.0 mmol) and ACVA (57 mg, 0.21 mmol) were dissolved in a mixed solvent of DMSO and water (9/1, *v*/*v*, 10 mL). The solution was deoxygenated by purging with nitrogen gas for 30 min, and then heated with an oil bath thermostated at 60 °C with stirring for 7 h. Subsequently, the reaction mixture was treated with 1 M NaOH (100 mL) at room temperature overnight. The polymer obtained was purified by dialysis against water for 15 days. Yield 3.41 g, 87.0%.

DMA (5.1 mL, 50 mmol) and ACVA (140 mg, 0.498 mmol) were dissolved in water (50 mL). The solution was deoxygenated by purging with nitrogen gas for 30 min, and then heated with an oil bath thermostated at 70 °C with stirring for 18 h. The polymer obtained was purified by dialysis against water for six days. Yield 3.96 g, 77.8%.

### 2.4. Preparation of N,N-Dimethylacrylamide/4-Vinylphenylboronic Acid Copolymer

DMA (4.1 mL, 40 mmol), VBA (59 mg, 0.40 mmol), and ACVA (112 mg, 0.399 mmol) were dissolved in DMF (40 mL). The solution was deoxygenated by purging with argon gas for 1 h, and then heated with an oil bath thermostated at 70 °C with stirring for 6 h. The polymer obtained was purified by dialysis against water for five days. Yield 3.01 g, 72.5%.

### 2.5. Formose Reaction in the Presence of Boronic Acid Compounds

A typical procedure of the formose reaction is described below. After pDMA/VBA (10 mg) was dissolved in an aqueous solution of formaldehyde (1 M, 2 mL), calcium hydroxide (18.8 mg, 0.240 mmol) was added to the mixture. The reaction mixture was warmed with a water bath thermostated at 60 °C with stirring. At predetermined reaction times, aliquots of the reaction mixture were taken to determine formaldehyde consumption by the acetylacetone method [38]. After a predetermined time, the reaction was quenched by adding 1 M HCl (0.24 mL). After the reaction mixture was treated with the ion exchenge resins for 30 min, the product was purified by dialysis against water (300 mL) for one day. The outer aqueous solution was concentrated under reduced pressure. After lyophilization of the solution, the product was obtained as pale brown solid. Yield 28 mg, 47%.

## 3. Results

### 3.1. Effect of Water Soluble Polymers on the Formose Reaction

**Figure 1:**
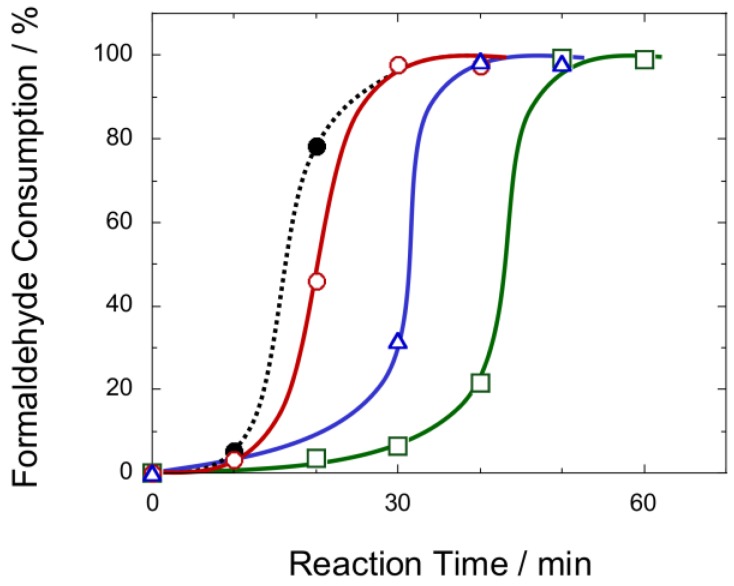
Time–conversion plots for the formose reaction carried out using 0.2 M formaldehyde and 40 mM calcium hydroxide at 60 °C in the absence (black circle, dotted line) and presence of 10 g·L^−1^ pNaSS (blue triangle), pVTAOH (green square), and pDMA (red circle). The curves are drawn as a guide for the eye.

Before investigation on the formose reaction in the presence of polymer-carrying boronic acid moieties, we started this study with an examination on the effect of water soluble polymers. We employed anionic poly(sodium 4-styrenesulfonate) (pNaSS), cationic poly(4-vinylbenzylammonium hydroxide) (pVTAOH), and nonionic poly(*N*,*N*-dimethylacrylamide) (pDMA) in this study. Figure 1 shows time–conversion plots for the formose reaction carried out using 0.2 M formaldehyde and 40 mM calcium hydroxide at 60 °C in the absence and presence of these polymers. In the absence of the polymers, the formaldehyde consumption increased up to a quantitative one within 30 min. However, the formose reaction was decelerated significantly in the presence of 10 g·L^−1^ pNaSS and pVTAOH, and the formaldehyde consumption became quantitative after ca. 40 and 50 min, respectively. In the presence of 10 g·L^−1^ pDMA, the formose reaction was slowed down only slightly, i.e., the formaldehyde consumption reached a quantitative one within 30 min. This observation indicates that pDMA does not considerably perturb the formose reaction.

### 3.2. Preparation of N,N-Dimethylacrylamide/4-Vinylphenylboronic Acid Copolymer

On the basis of the observations described above, we have chosen a copolymer of DMA and VBA (pDMA/VBA) to study the effect of polymer-carrying boronic acid moieties on formose reaction in this study. The copolymer was prepared by radical copolymerization of DMA and VBA in DMF using ACVA as initiator. The pDMA/VBA copolymer was purified by dialysis against water for five days. The VBA content in the copolymer was determined to be ca. 2 mol% by ^1^H NMR. The *M*_n_ and *M*_w_/*M*_n_ values were evaluated to be 3.58 × 10^5^ and 1.58, respectively, by SEC. Thus, a single chain carries ca. 70 boronic acid moieties in average. It is likely that the distribution of VBA units on the polymer chain is practically random.

### 3.3. Effect of the N,N-Dimethylacrylamide/4-Vinylphenylboronic Acid Copolymer

**Figure 2:**
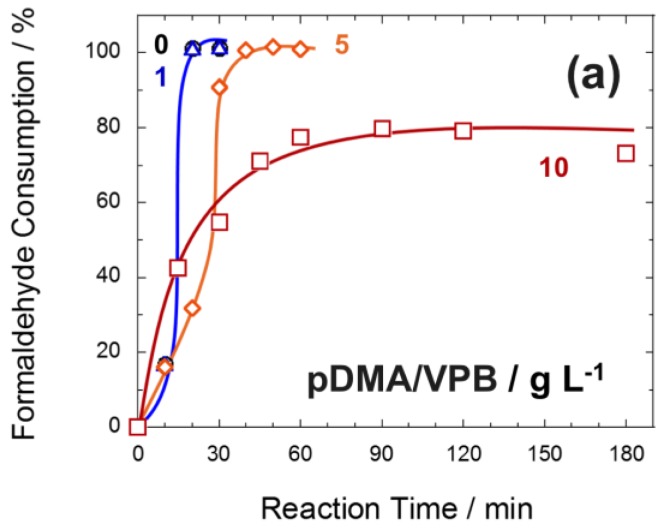

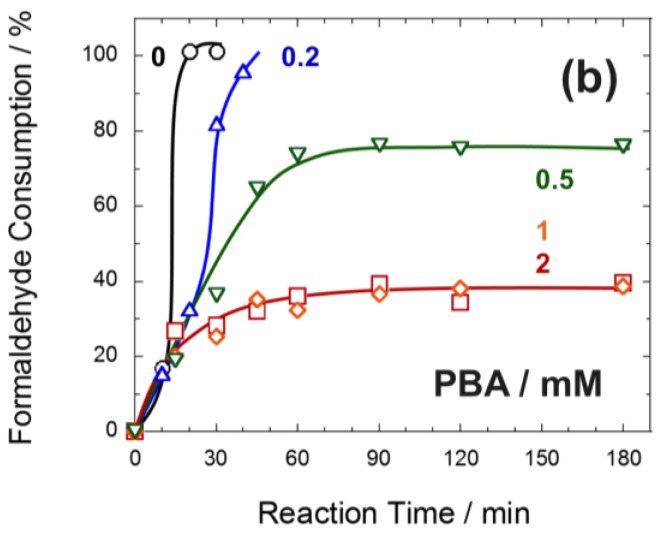
Time–conversion plots for the formose reaction carried out using 1 M formaldehyde and 120 mM calcium hydroxide at 60 °C in the presence of 0 (black circle), 0.2 (blue triangle), 0.5 (green inverted triangle), 1 (orange diamond), and 2 mM (red square) PBA (**a**) and 0 (black circle), 1 (blue triangle), 5 (orange diamond), and 10 g·L^−1^ (red square) pDMA/VBA (**b**). The same colors indicate the same concentrations of boronic acid moieties. The curves are drawn as a guide for the eye.

Since the formose reaction did not proceed in the presence of NaPB or pNaSS/NaVB under the reaction conditions in our previous study, a co-catalyst, i.e., fructose or glyceraldehyde, was necessary for conversion of formaldehyde. In this study, we have used the reaction conditions under which the reaction proceeded without any co-catalyst. We carried out the formose reaction using 1 M formaldehyde and 120 mM calcium hydroxide. Figure 2a indicates the time–conversion plots in the absence and presence of varying concentrations of pDMA/VBA. In the absence of pDMA/VBA, the formaldehyde consumption increased rapidly up to a quantitative one within 20 min. In the presence of 1 g·L^−1^ pDMA/VBA, the time–conversion plots were the same as those for the conventional formose reaction. In the presence of 5 g·L^−1^ pDMA/VBA, slight retardation of the reaction was observed, but the formaldehyde consumption became quantitative after 40 min. At 10 g·L^−1^ pDMA/VBA, significant retardation was not observed, but the formaldehyde consumption leveled off at ca. 80% after 60 min. These observations indicate that polymer-carrying boronic acid moieties retard the formose reaction because DMA units have almost no effect. Similarly, in the case of 0.2 mM PBA, the formose reaction was slightly decelerated, and the formaldehyde consumption increased up to a quantitative one after 40 min. In the presence of 0.5 mM PBA, the formaldehyde consumption leveled off at ca. 80% after 60 min. At 1 and 2 mM PBA, the formaldehyde consumption was saturated at ca. 40%. These observations confirm that PBA retards the formose reaction. A solution of 5 g·L^−1^ pDMA/VBA contains ca. 1 mM boronic acid moieties. It should be noted here that 1 mM PBA exhibits a stronger effect of retardation than does 5 g·L^−1^ pDMA/VBA. This observation indicates that low molecular weight boronic acid moieties have a stronger effect of retardation than do polymer-carrying ones presumably because of a lower effective concentration of polymer-carrying moieties.

### 3.4. Confirmation of the Formation of Boronic Acid Esters in Formose Reaction Mixtures

**Figure 3:**
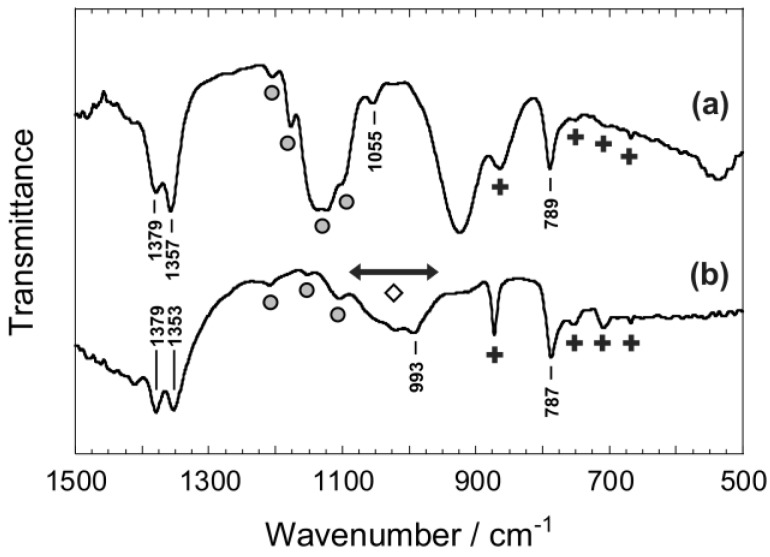
IR spectra for the reaction mixture containing 1 M formaldehyde, 120 mM calcium hydroxide, and 2 mM PBA before (**a**) and after heating at 60 °C for 60 min (**b**). The measurements were carried out after evaporation of the solvent by air blowing on a diamond prism loaded in the ATR attachment. Symbols (cross and circle) denote the absorption bands assignable to calcium hydroxide and formaldehyde, respectively.

It is likely that the retardation effect of boronic acid moieties in formose reaction is caused by the formation of boronic acid esters. To confirm the formation of boronic acid esters, IR spectra were recorded for mixtures before and after the reaction (Figure 3). Figure 3a,b contain absorption bands ascribable to calcium hydroxide at 668, 710, 750, and 872 cm^−1^ (see Figure S1 in Supplementary Materials). In Figure 3a, absorption bands at 1055, 1357, and 1379 cm^−1^ are assignable to the B–O–H deformation, B–O stretching, and B–O and B–C stretching vibrations, respectively [39,40]. Figure 3a also exhibits an absorption band due to the aromatic C–H deformation vibration at 789 cm^−1^ and a board absorption band assignable to formaldehyde in the region of 1000–1200 cm^−1^. In the IR spectrum for the reaction mixture (Figure 3b), absorption bands at 787, 1353, and 1379 cm^−1^ are assignable to the aromatic C–H deformation, B–O stretching, and B–O and B–C stretching vibrations, respectively. It is noteworthy that Figure 3b does not exhibit any absorption band due to B–O–H deformation at ca. 1055 cm^−1^ whereas it contains a absorption band due to the B–O–H in quaternized boronic acid esters at 993 cm^−1^ [41] and a broad absorption band due to the C–O stretching vibration in the region of 950–1100 cm^−1^. These observations indicate that as the formose reaction proceeds in the presence of PBA, the free boronic acid is consumed to form esters.

### 3.5. Characterization of Formose Reaction Products

**Figure 4:**
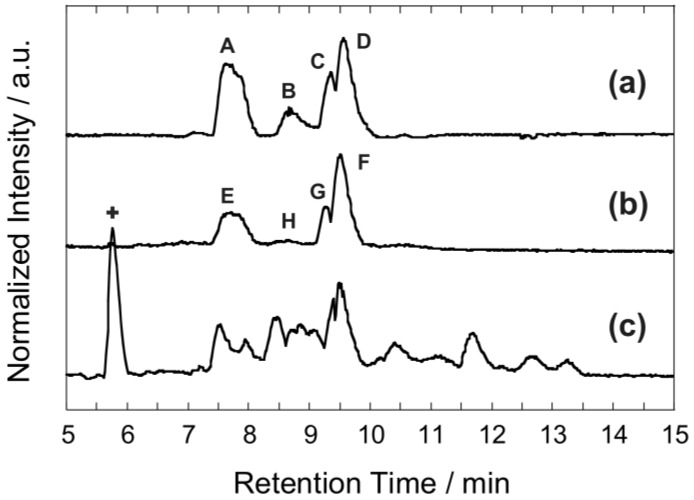
Mass chromatograms for the products of the formose reaction carried out using 1 M formaldehyde and 120 mM calcium hydroxide at 60 °C in the presence of 10 g·L^−1^ pDMA/VBA (**a**) and 0.5 mM PBA (**b**), and in their absence (**c**). The reaction time was 180 (**a**,**b**) or 20 min (**c**). The *m*/*z* range of 60–250 was detected.

The formose reaction products were characterized by LC-MS. The products were purified by treatment of the reaction mixtures with the ion-exchange resins followed by dialysis against pure water. Figure 4a,b display mass chromatograms for the products of the formose reaction carried out in the presence of pDMA/VBA and PBA, respectively. For reference, the mass chromatogram for the product without any boronic acid compound is indicated in Figure 4c. This figure indicates a number of signals in the elution time region of 7–14 min, indicative of the formation of a complicated mixture in the conventional formose reaction. On the other hand, as can be seen in Figure 4a,b, the mass chromatograms for the products of formose reaction in the presence of pDMA/VBA and PBA exhibit four major signals at 7.7, 8.7, 9.3, and 9.6 min. These chromatograms do not contain any signals in the elution time region of 10–14 min, indicating that the selectivity of the formose reaction is somehow enhanced.

**Figure 5:**
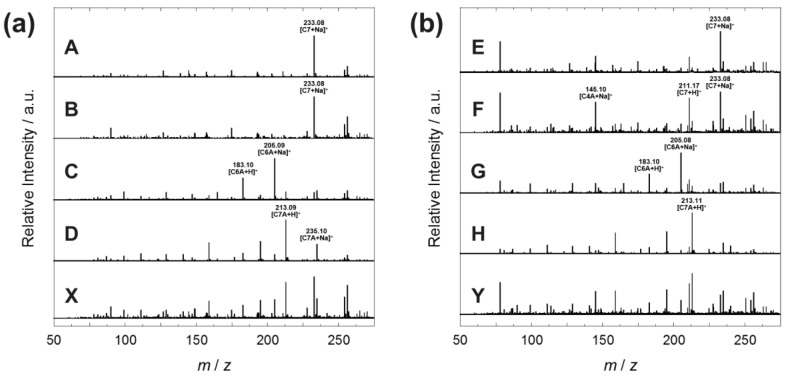
Mass spectra obtained in the LC-MS measurements for the products of the formose reaction carried out using 1 M formaldehyde and 120 mM calcium hydroxide at 60 °C for 180 min in the presence of 10 g·L^−1^ pDMA/VBA (**a**) and 0.5 mM PBA (**b**). Spectra A–H were recorded at the maxima of signals A–H in Figure 4. Spectra X and Y are the total mass spectra recorded in the region of 7–14 min in Figure 4a,b, respectively. C*n* and C*n*A denote *n*-carbon monosaccharide and sugar alcohol, respectively.

Figure 5 compares the mass spectra for the four major signals in Figure 4a,b. As a eference, the mass spectrum for the product of the conventional formose reaction is shown in Figure S3 in the Supplementary Materials. The spectrum indicates a number of signals at *m*/*z* = 145.06, 153.09, 175.07, 183.10, 203.10, 213.12, and 232.08 assignable to four- to seven-carbon sugar alcohols, as well as six-carbon monosaccharides (for assignments see Figure S3 in Supplementary Materials). These signals are indicative of the formation of a complicated mixture. Figure 5a,b show the mass spectra at the maxima of four major signals in Figure 4a,b for the products of the formose reaction in the presence of pDMA/VBA and PBA, respectively. Figure 5a,b indicate that the formose reaction yields, selectively, six- and seven-carbon monosaccharides and sugar alcohols in the presence of pDMA/VBA and PBA.

**Figure 6:**
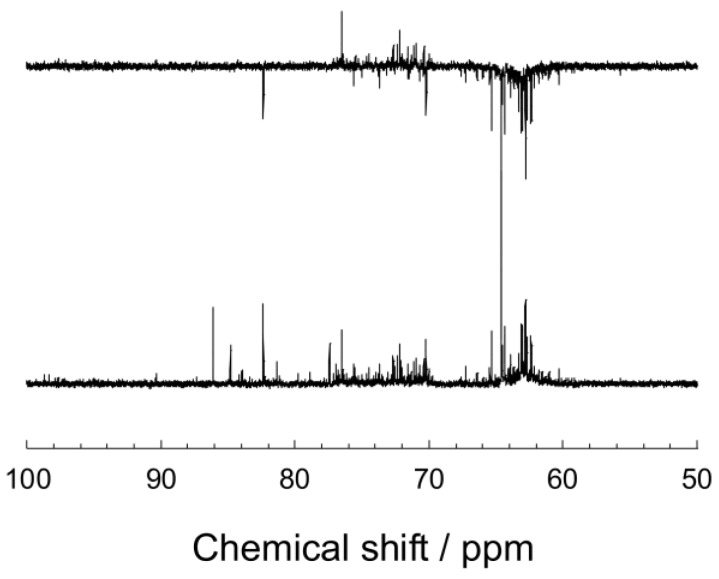
^13^C NMR (**lower**) and DEPT-135 spectra (**upper**) for the product of the formose reaction carried out using 1 M formaldehyde and 120 mM calcium hydroxide at 60 °C for 60 min in the presence of 5 g·L^−1^ pDMA/VBA.

The structure of products was also characterized by NMR spectroscopy. As an example, Figure 6 shows ^13^C NMR and DEPT-135 spectra for the product of formose reaction in the presence of pDMA/VBA. The ^13^C NMR spectrum shows a number of signals, indicating that the product contains some isomers. The DEPT-135 spectrum contains positive and negative signals ascribed to methine and methylene carbons, respectively. It is noteworthy that there are significant signals ascribable to quaternary carbons in the region of 70–90 ppm in the ^13^C NMR spectrum, which are not observed in the DEPT-135 spectrum. This observation indicates that the products contain branched monosaccharides and sugar alcohols. The formation of branched products was confirmed by HSQC and HMBC spectra (Figures S4 and S5 in Supplementary Materials).

## 4. Discussion

**Figure 7:**
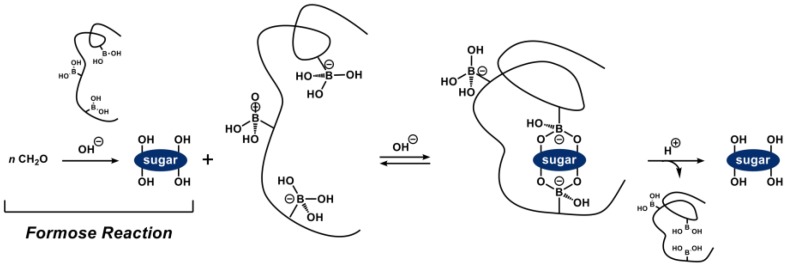
Conceptual illustration of formose reaction in the presence of a boronic acid polymer, in which the polymer stabilizes the product via ester formation, leading to an enhanced selectivity.

Here we discuss why boronic acid compounds somehow enhance the selectivity of formose reaction products. As described in the introduction part, formose reaction proceeds via some elemental steps, including acyloin condensation, aldol and retro-aldol reactions, aldose–ketose isomerization, and the Cannizzaro reaction. Thus, it is known that formose reaction provides favorably branched monosaccharides and sugar alcohols [24,25,42]. On the other hand, boronic acid compounds form esters with the formose reaction products. Since added boronic acid compounds significantly retard the formose reaction, the formation of boronic acid esters protect the products against further reactions. The stability of boronic acid esters depends on the relative orientation of two hydroxy groups, i.e., the species of monosaccharides and sugar alcohols. It is also known that boronic acid esters of a five- or six-membered ring are rather stable [43]. It is, thus, likely that formose reaction in the boronic acid compounds produces favorably six- and seven-carbon monosaccharides and the corresponding sugar alcohols, which can form rather stable esters of a five- or six-membered ring (Figure 7).

It has been reported that two boronic acid moieties of a specific relative location form ester with monosaccharides showing an enhanced selectivity [36]. Thus, molecularly imprinted polymers [44,45] or cross-linked dendrimers [46], in which two boronic acid moieties are located appropriately, are promising to give a higher selectivity of the formose reaction product.

## Supplementary Materials

The following are available online at www.mdpi.com/2073-4360/9/11/549/s1. Figure S1. IR spectra for the standard samples and the reaction mixtures: A mixture of 1 M formaldehyde and 120 mM calcium hydroxide in water (a), a mixture of 120 mM calcium hydroxide and 2 mM PBA in water (b), PBA (c), the mixtures containing 1 M formaldehyde, 120 mM calcium hydroxide, and 2 mM PBA before (d) and after formose reaction at 60 °C for 60 min (e). The measurements were carried out after evaporation of the solvent by air blowing on a diamond prism loaded in the ATR attachment; Figure S2. IR spectrum computationally calculated for the ester of quaternized diphenylboronic acid with fructose using a Gaussian 09W software with the following settings: calculation type, frequency; calculation method, RHF; basis set, 3–21 G (upper panel), and a snapshot of the three-dimensional vibrational structure responsible for the absorption band at 974 nm^−1^ (lower panel); Figure S3. The mass spectrum recorded in LC-MS measurements for the region of 7–14 min in Figure 4c, corresponding to the products of formose reaction carried out using 1 M formaldehyde and 120 mM calcium hydroxide at 60 °C for 20 min in the absence of the boronic acid compounds; Figure S4. HSQC spectrum for the product of formose reaction carried out using 1 M formaldehyde and 120 mM calcium hydroxide at 60 °C for 60 min in the presence of 5 g·L^−1^ pDMA/VBA; Figure S5. HMBC spectrum for the product of formose reaction carried out using 1 M formaldehyde and 120 mM calcium hydroxide at 60 °C for 60 min in the presence of 5 g·L^−1^ pDMA/VBA; Video S1. The three-dimensional vibrational structure responsible for the absorption band at 974 nm^−1^ in the calculated spectrum (Figure S2).
