# Supplementary material for: Formose Reaction Controlled by a Copolymer of N,N-Dimethylacrylamide and 4-Vinylphenylboronic Acid
Source: Polymers (Basel). 2017 Oct 25;9(11):549. doi: 10.3390/polym9110549 (PMC6418552; doi:10.3390/polym9110549)
Supplement: Supplementary file 1 [file polymers-09-00549-s001.zip › si_polymers-236000.pdf]

Supplementary Materials for

Formose Reaction Controlled by a Copolymer of *N*,*N*-Dimethylacrylamide and 4-Vinylphenylboronic Acid

Tomohiro Michitaka [1], **Toru Imai** [1], **and Akihito Hashidzume** [1,*]

[1] Department of Macromolecular Science, Graduate School of Science, Osaka University; hashidzume@chem.sci.osaka-u.ac.jp

* Correspondence: hashidzume@chem.sci.osaka-u.ac.jp; Tel.: +81-6-6850-8174

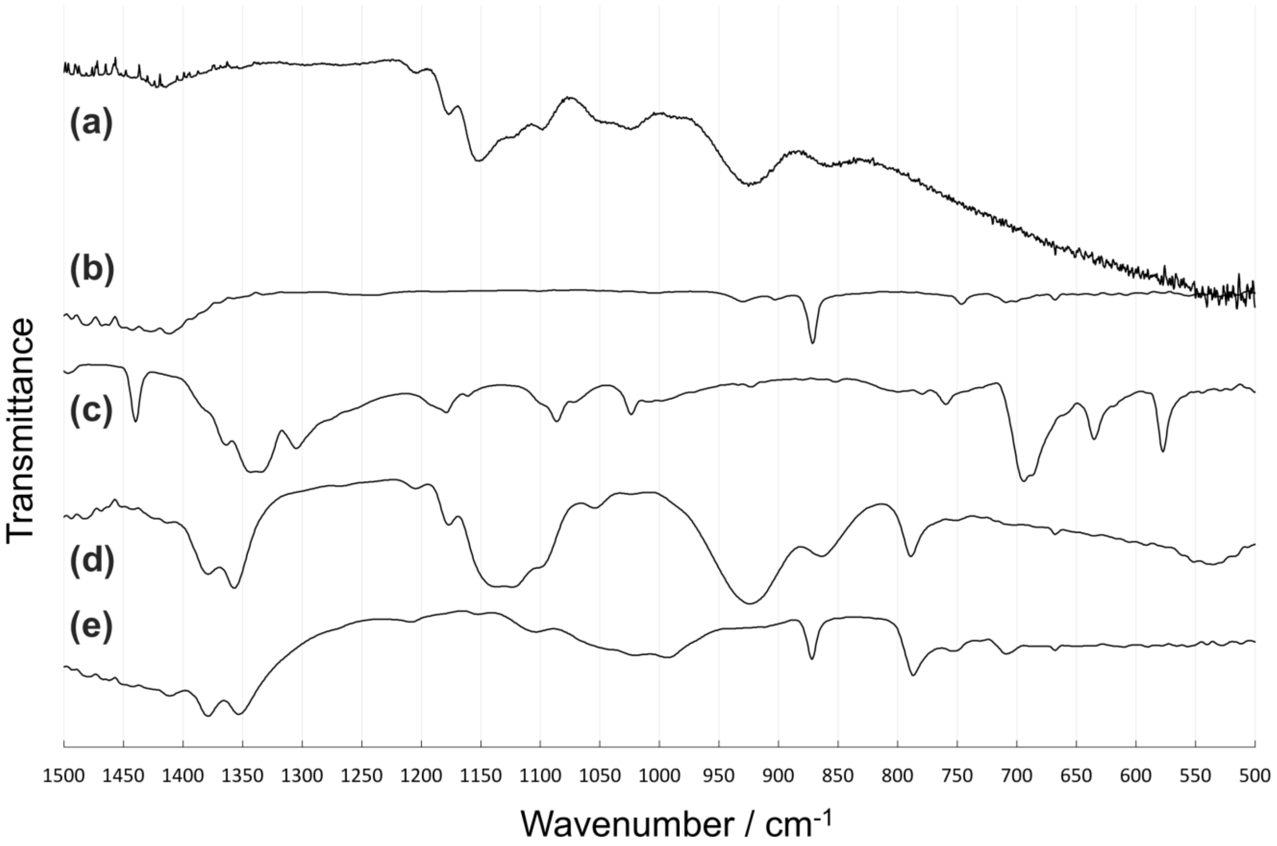

Figure S1. IR spectra for the standard samples and the reaction mixtures: A mixture of 1 M formaldehyde and 120 mM calcium hydroxide in water (a), a mixture of 120 mM calcium hydroxide and 2 mM PBA in water (b), PBA (c), the mixtures containing 1 M formaldehyde, 120 mM calcium hydroxide, and 2 mM PBA before (d) and after formose reaction at 60 °C for 60 min (e). The measurements were carried out after evaporation of the solvent by air blowing on a diamond prism loaded in the ATR attachment.

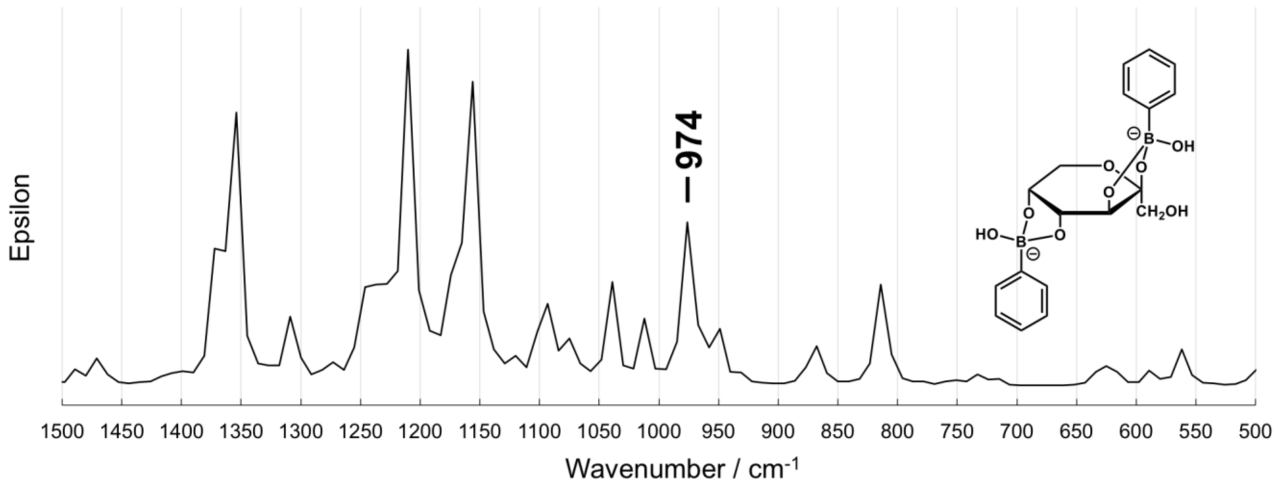

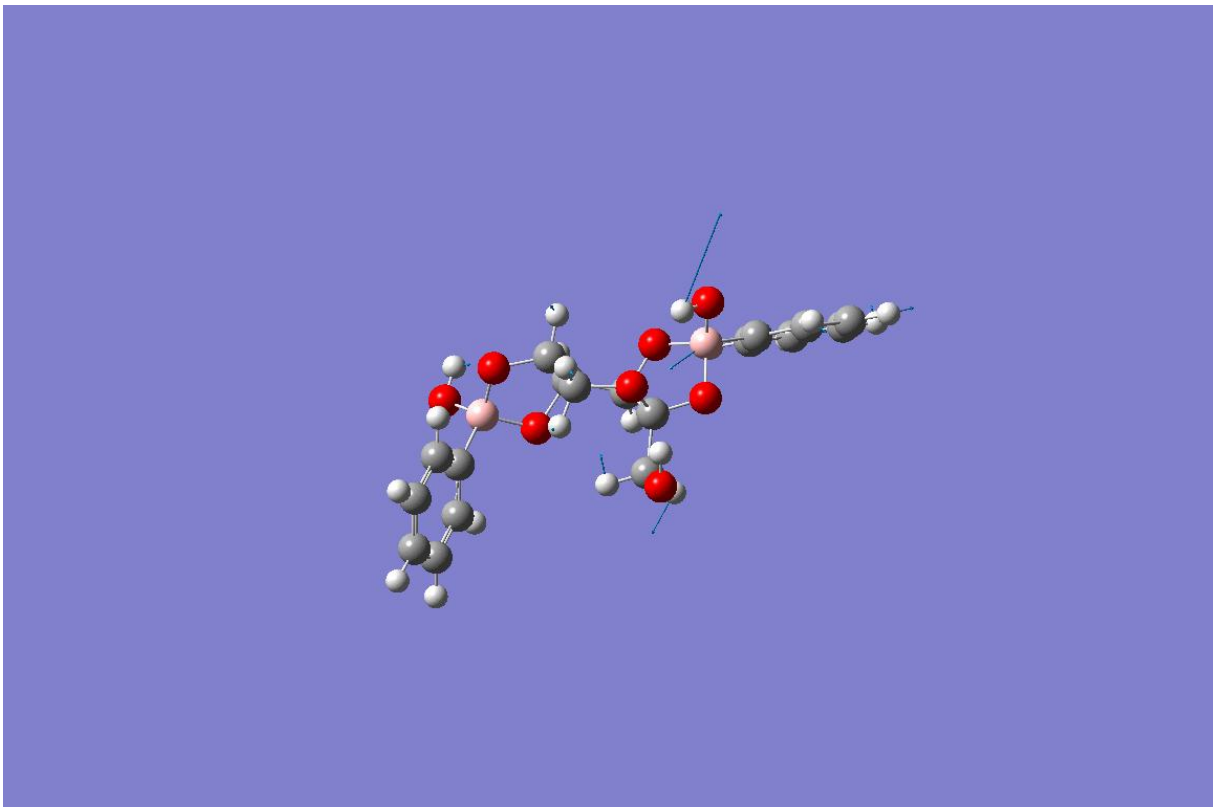

Figure S2. IR spectrum computationally calculated for the ester of quaternized diphenylboronic acid with fructose using a Gaussian 09W software with the following settings: Calculation type, Frequency; calculation method, RHF; basis set, 3–21G (upper) and a snapshot of the three-dimensional vibrational structure responsible for the absorption band at 974 nm^{-1} (lower).

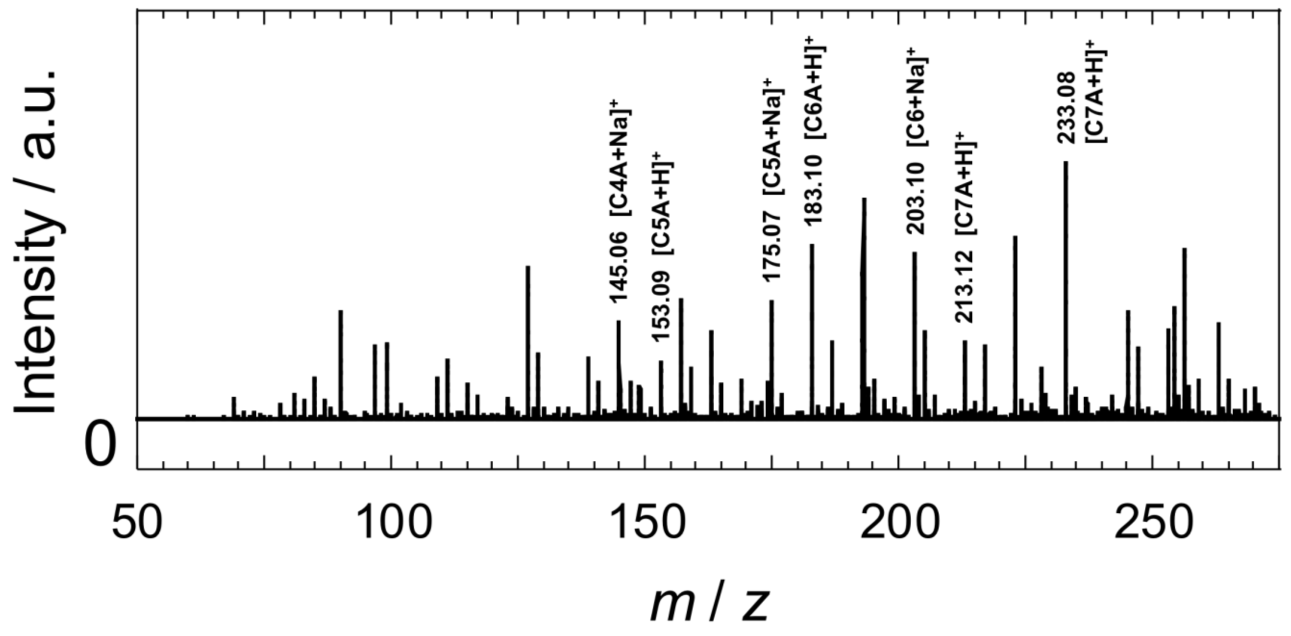

Figure S3. Mass spectrum recorded in LC-MS measurements for the region of 7 – 14 min in Figure 4 (c), corresponding to the products of formose reaction carried out using 1 M formaldehyde and 120 mM calcium hydroxide at 60 °C for 20 min in the absence of the boronic acid compounds.

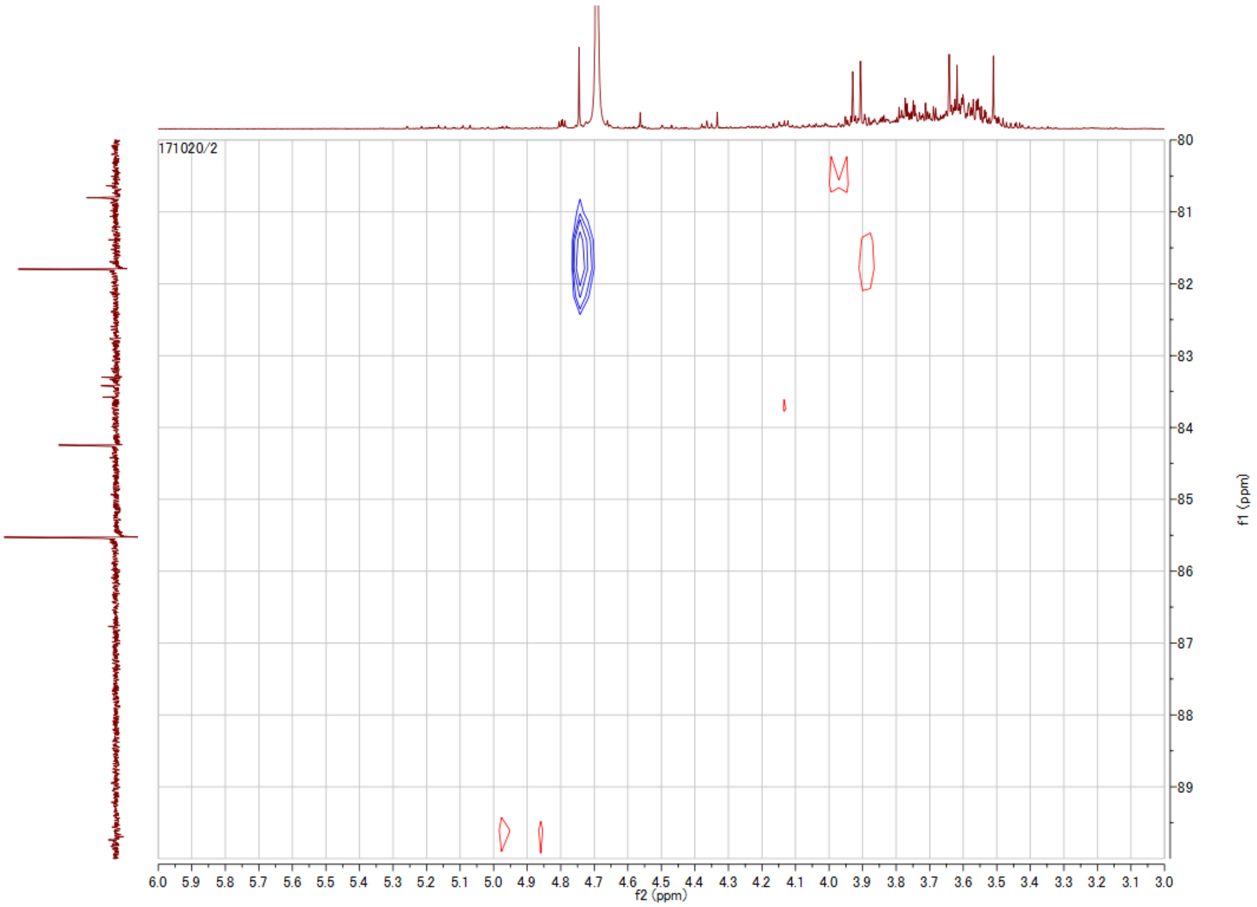

Figure S4. HSQC spectrum for the product of formose reaction carried out using 1 M formaldehyde and 120 mM calcium hydroxide at 60 °C for 60 min in the presence of 5 g L^{-1} pDMA/VBA. The signals due to quaternary carbons in the ppm range of 80 – 86 ppm do not show any correlations with ^{1}H signals.

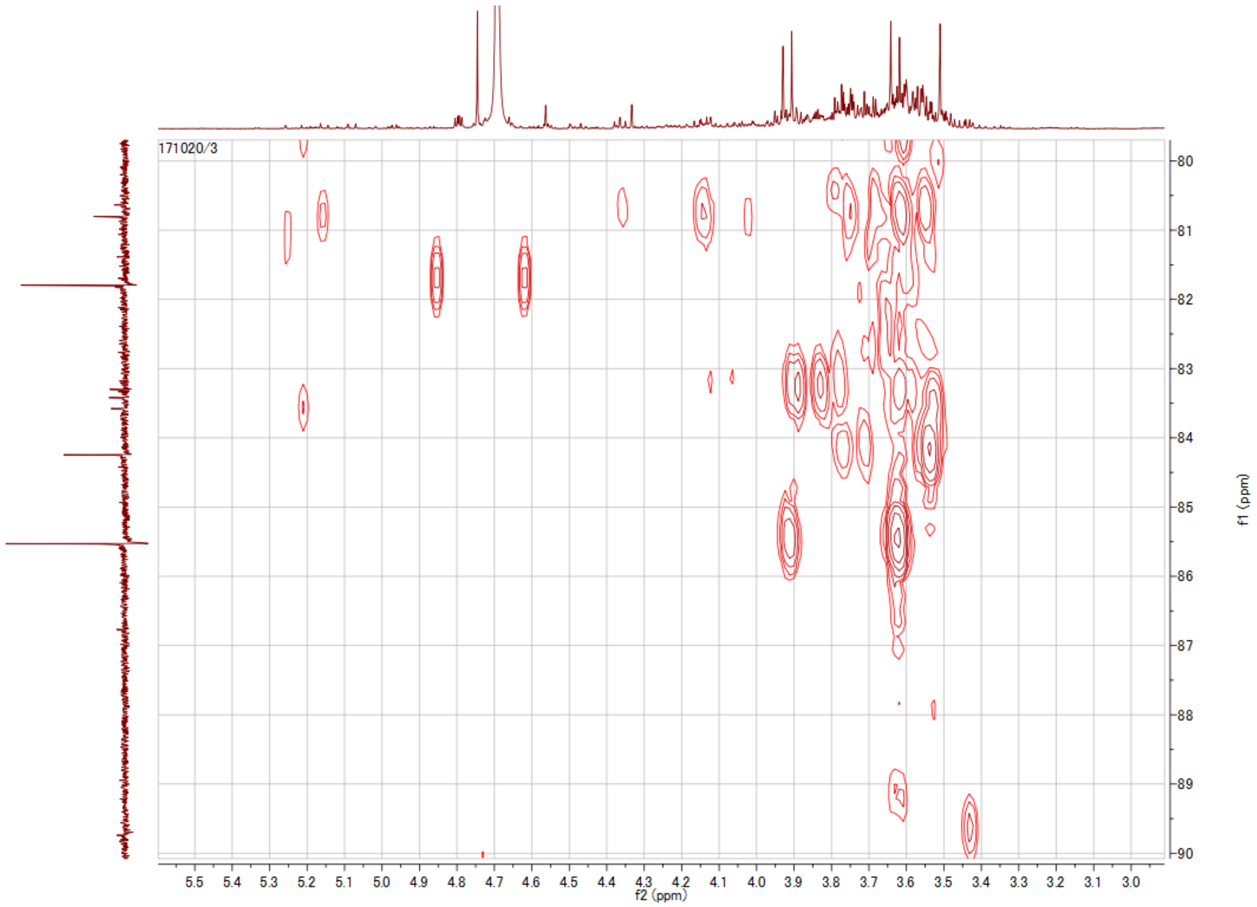

Figure S5. HMBC spectrum for the product of formose reaction carried out using 1 M formaldehyde and 120 mM calcium hydroxide at 60 °C for 60 min in the presence of 5 g L^{-1} pDMA/VBA. The signals due to quaternary carbons in the ppm range of 80 – 86 ppm show correlations with ^{1}H signals.
