# Supplementary figures and images for: Formose Reaction Controlled by a Copolymer of N,N-Dimethylacrylamide and 4-Vinylphenylboronic Acid
Source: Polymers (Basel). 2017 Oct 25;9(11):549. doi: 10.3390/polym9110549 (PMC6418552; doi:10.3390/polym9110549)

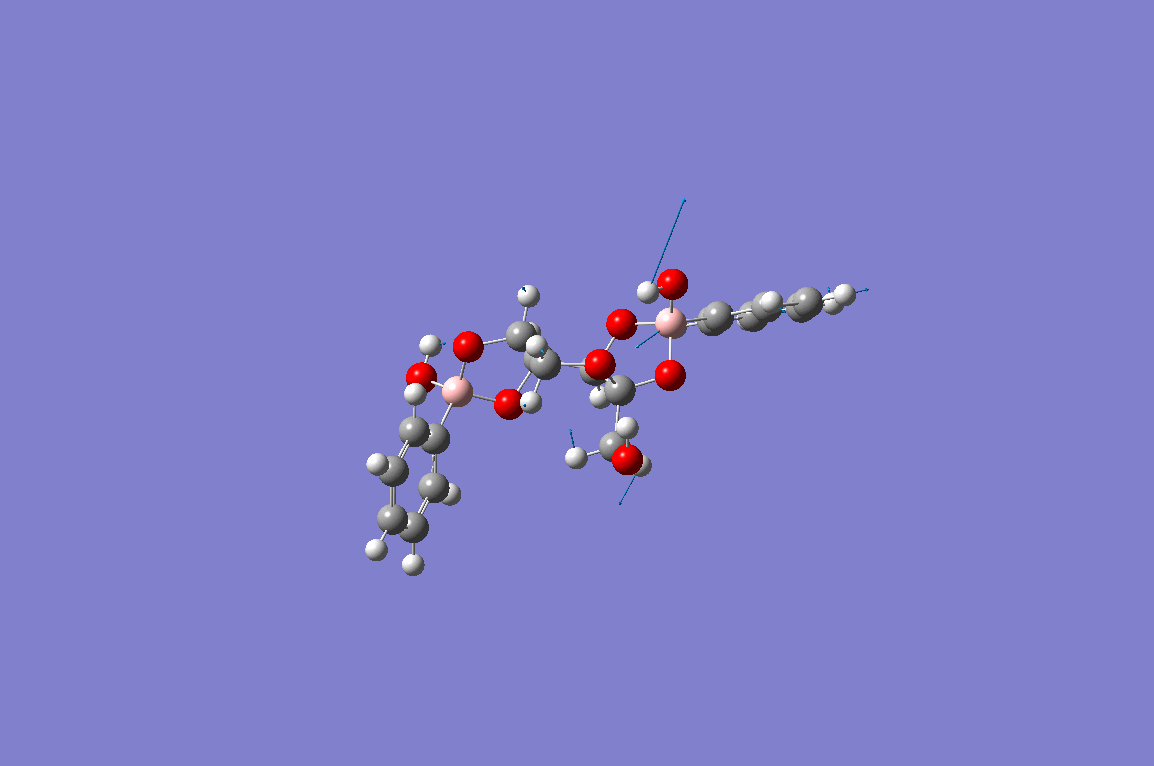

Supplement: Supplementary file 1 [file polymers-09-00549-s001.zip › vs1-polymers-236000.gif]
